# COVID-19 lessons to protect populations against future pandemics by implementing PPPM principles in healthcare

**DOI:** 10.1007/s13167-023-00331-7

**Published:** 2023-07-14

**Authors:** Cuihong Tian, Lois Balmer, Xuerui Tan

**Affiliations:** 1 Clinical Research Center, First Affiliated Hospital of Shantou University Medical College, Shantou, 515041 Guangdong China; 2 Department of Cardiology, First Affiliated Hospital of Shantou University Medical College, Shantou, 515041 Guangdong China; 3 Center for Precision Health, Edith Cowan University, Perth, WA 6027 Australia

**Keywords:** COVID-19, SARS-CoV-2, Case fatality rate (CFR), Virus variant, Asymptomatic infection, Sequelae, Excess mortality, Predictive preventive personalised medicine (PPPM), Public Health Emergency of International Concern (PHEIC), Socio-economic burden, Health policy, Individual outcomes, Future pandemics, Population protection

## Abstract

The coronavirus disease 2019 (COVID-19) pandemic has continued for more than 3 years, placing a huge burden on society worldwide. Although the World Health Organization (WHO) has declared an end to COVID-19 as a Public Health Emergency of International Concern (PHEIC), it is still considered a global threat. Previously, there has been a long debate as to whether the COVID-19 emergency will eventually end or transform into a more common infectious disease from a PHEIC, and how should countries respond to similar pandemics in the future more time-efficiently and cost-effectively. We reviewed the past, middle and current situation of COVID-19 based on bibliometric analysis and epidemiological data. Thereby, the necessity is indicated to change the paradigm from reactive healthcare services to predictive, preventive and personalised medicine (PPPM) approach, in order to effectively protect populations against COVID-19 and any future pandemics. Corresponding measures are detailed in the article including the involvement of multi-professional expertise, application of artificial intelligence, rapid diagnostics and patient stratification, and effective protection, amongst other to be considered by advanced health policy.

## Introduction

### COVID-19-associated global socio-economic burden

As of May 24, 2023, approximately 766 million people were infected with coronavirus disease 2019 (COVID-19) and 6.94 million died worldwide [1]. The COVID-19 pandemic has caused a depletion of social resources as well as anxiety and chronic fatigue amongst citizens. The health system, cultural tourism industry, export transactions, abroad education and catering service were all overwhelmed. Statistically, gross domestic product (GDP) decreased by 6.7% in 2020 globally due to the prevalence of COVID-19 [2]. Although most countries have lifted restrictions much earlier than the date of May 5, 2023, when WHO declared the end of COVID-19 as a public health emergency, it can still be considered a global threat [3]. Previously, there has been a long debate as to whether the COVID-19 pandemic will eventually end or transform into a more common infectious disease from the status of Public Health Emergency of International Concern (PHEIC), and how should actually countries respond to a similar pandemic in a more time-efficient and cost-effective way in the future. With this article, we would like to contribute to this debate on the bases of accumulated data.

### Challenges of combatting pandemics

Predictive, preventive and personalised medicine (PPPM) is an advanced paradigm in healthcare aiming at effective protection of populations against both communicable and non-communicable disorders in primary care and at improved individual outcomes in secondary care utilising predictive diagnosis, targeted prevention and treatments tailored to individualised patient profiles [4]. This allows healthcare providers to intervene early, often before the patient has any symptoms, and to prevent or delay the onset of the disease. PPPM is beneficial to healthcare by improving patient outcomes, reducing healthcare costs and enhancing the efficiency of healthcare delivery, which is applicable not only to chronic non-infectious diseases’ management, e.g. diabetes [5] and cancers [6, 7], but also to infectious disease control, such as COVID-19 [8]. However, how to maximise the role of PPPM in preventing a pandemic is an essential prerequisite to early identification, detection and quick response of future PHEIC!

### Working hypothesis

It is hypothesised that a timely and effective application of predictive diagnostics, targeted prevention and personalisation of treatment algorithms to a great extent may protect populations against future pandemics and devastating outcomes. To prove the working hypothesis, in-depth analyses of the COVID-19-related data are provided in the current article.

### Bibliometric analysis of COVID-19

Based on Citexs, a professional, free and open data analysis platform, and PubMed literature database, we reviewed the literature related to COVID-19 and analysed the historical trend of COVID-19 to the current year. We summarised the developmental history of COVID-19 and analysed the change of hot keywords, and then visualised the results via a systematic review. Afterwards, we excavated and analysed the strategies that need to be improved, to provide baseline information for managing the future similar pandemics like COVID-19.

**Fig. 1:**
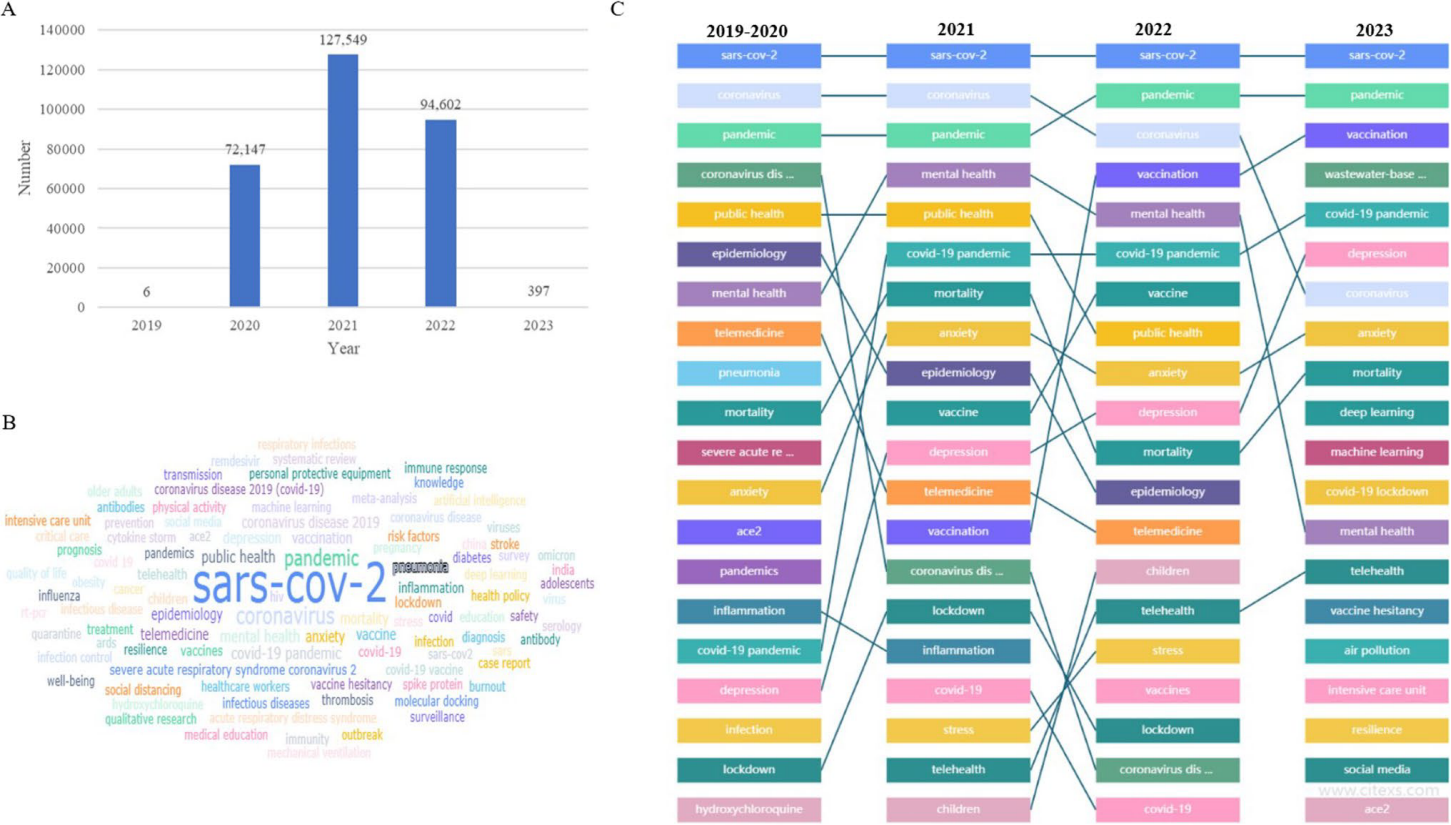
Bibliometric analysis visualised by Citexs platform. **A** The number of articles related to COVID-19 each year. The histogram shows that there are 6, 72,147, 127,549, 94,602 and 397 English articles retrieved from the PubMed database in the year 2019, 2020, 2021, 2022 and 2023, respectively. **B** Word cloud analysis based on the frequencies of COVID-19-related keywords. The word cloud is a useful tool to identify key themes and keywords within large sets of text data. The keywords related to COVID-19 were visualised by word cloud analysis, in which the size of each keyword represents its frequency or importance within the articles. **C** The frequency change of COVID-19-related keywords in each year. For example, the frequency of “SARS-CoV-2” is always kept at the highest level. The frequencies of the keywords “depression” and “vaccination” increased significantly from 2019 to 2023, revealing that they were gradually valued

A total of 294,701 English articles were retrieved from the PubMed database with the index terms “COVID-19 or coronavirus disease 2019 or SARS-CoV-2”. The number of published articles in 2020 grew considerably, indicating that COVID-19 became a hot topic and attracted keen research interest from scholars worldwide. After reaching the peak of 127,549 articles in 2021, the number of COVID-19-related articles reduced to 94,602 articles in 2022, revealing that the topic of COVID-19 gradually faded in 2022 (Fig. 1A).

Amongst these articles, the top five keywords in terms of high frequency are “SARS-CoV-2”, “coronavirus”, “pandemic”, “mental health” and “public health” (Fig. 1B). Figure 1C shows the heat ranking and frequency changes of COVID-19-related keywords in each year from 2019 to 2023. For example, the frequency of the word “vaccination” increased year by year, which revealed that (1) experts devoted themselves to developing COVID-19 vaccines in 2021; (2) countries were calling for individuals to vaccinate in 2022; and (3) the rate of vaccination grew considerably in early 2023. Therefore, according to the number of published articles, the change of keywords and the pandemic trend of COVID-19, we divided the COVID-19 timeline into the following three-time phases: initial (2019–2020), middle (2021) and current (2022–2023).

### The initial phase (2019–2020)

In December 2019, several pneumonia cases with a history of exposure to the Huanan Seafood Wholesale Market were first reported in Wuhan, China. This infectious disease was officially named *coronavirus disease 2019 (COVID-19)* by the World Health Organization (WHO) on February 11, 2020 [9], and was regarded as a PHEIC on February 12, 2020 [10]. COVID-19 had spread to 114 countries, resulting in 118,000 cases and 4291 deaths in a relatively short time by March 10, 2020. On March 11, 2020, WHO declared that COVID-19 should be regarded as a “pandemic” [11]. According to the real-time official data of WHO, as of 4:11 pm Central European Summer Time (CEST) on December 31, 2020, the number of confirmed COVID-19 cases has reached 81.47 million including 1.79 million deaths globally [1].

**Fig. 2:**
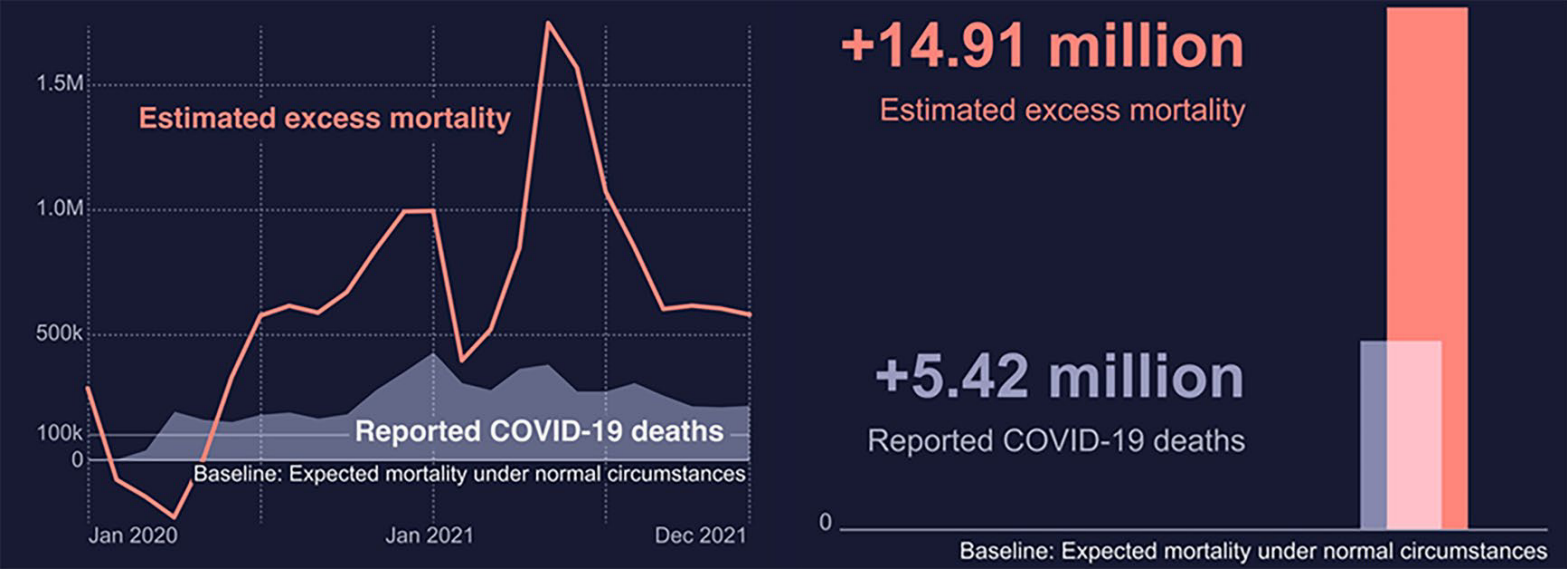
The global excess mortality and reported deaths. The zero at baseline represents the expected number of deaths according to the existing average mortality data from 2015 to 2019. The shaded area represents the number of cases that died of COVID-19 reported worldwide. The red line represents the estimated excess mortality, i.e. the additional people who died more than the expected number of deaths. The red number depicts the total excess mortality between January 1, 2020, and December 31, 2021. The data are available at https://www.who.int/data/stories/global-excess-deaths-associated-with-covid-19-january-2020-december-2021/

Human health and healthcare system were overwhelmed rapidly and disastrously at the early stage of COVID-19 pandemic due to the increased cases. The majority of health resources were being deployed to support patients with COVID-19, whilst other patients with chronic diseases were allocated few resources. Evidence suggested that during the first 3 months of the COVID-19 outbreak (the first quarter of 2020), the surging deaths in Wuhan were not only from COVID-19 but from other non-infectious diseases such as cardiovascular disease, cancer, diabetes and suicide [12]. Excess mortality, defined as the number of deaths in the period of pandemic relative to that expected in the normal period and used to evaluate the true mortality burden of COVID-19, considerably grew by about one million from January to December 2020 globally, compared with the average mortality from 2015 to 2019 (Fig. 2).

A drastic anti-COVID-19 campaign started around the world consecutively. Countries around the world responded cautiously to the outbreak of the COVID-19 pandemic. There were no approved medicines for COVID-19 treatment. Cutting off the transmission route of COVID-19 was the key strategy applied by almost all countries in the initial phase of COVID-19, including hand hygiene, wearing masks, social distancing and quarantine [13]. The government of China took the transmission very seriously and announced the lockdown of Wuhan on Chinese New Year’s Eve 2019. With a 76-day closure of departures from airports, railway stations and expressways, as well as delayed orientation and instigated online education, China wanted to minimise losses due to the pandemic. The United States of America (USA) issued a “global Level 4 health advisory” on March 19, 2020, advising Americans to avoid all international travel [14].

### The middle phase (2021)

**Fig. 3:**
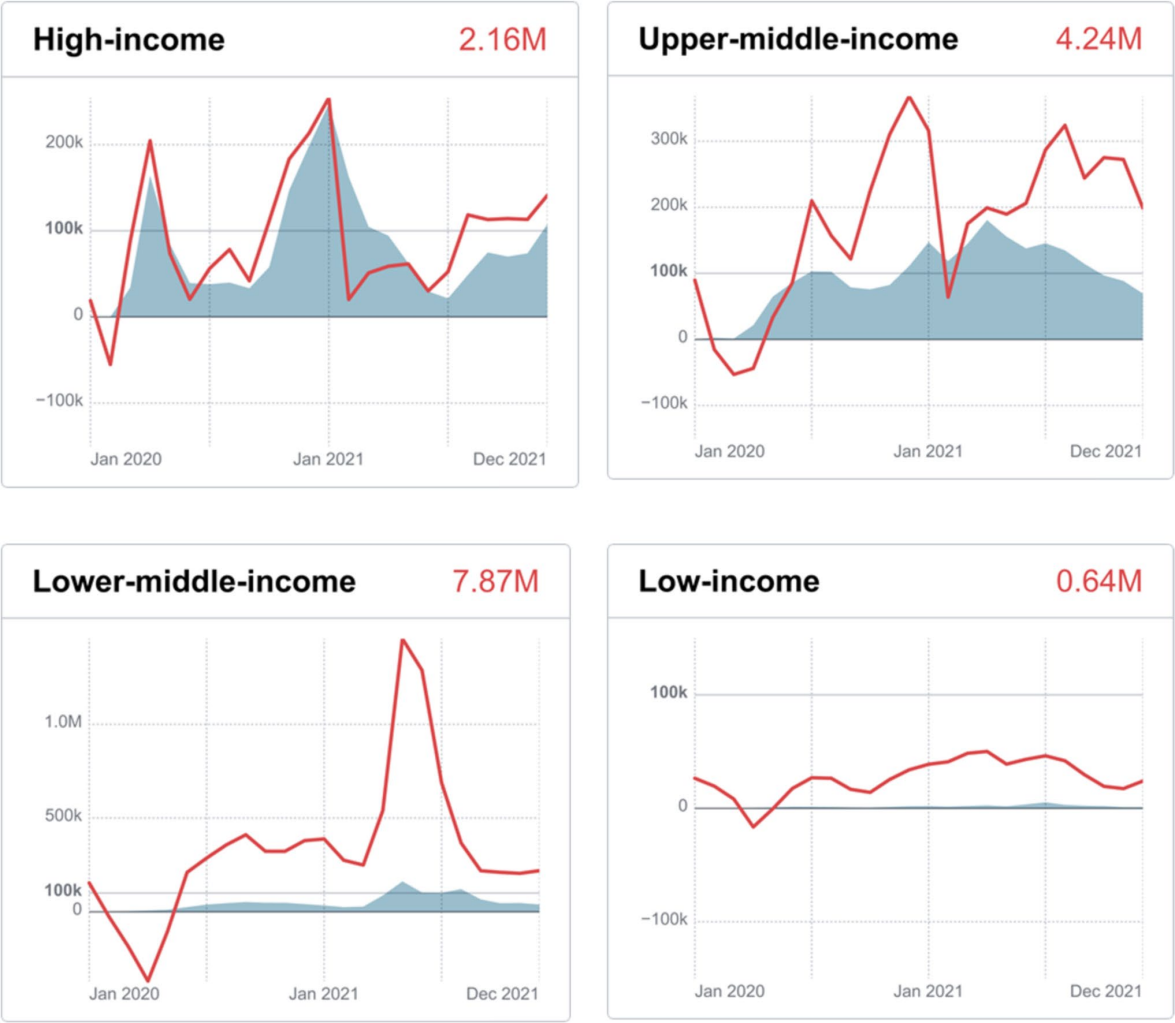
The excess mortality associated with COVID-19 in different World Bank income groups. The zero at baseline represents the expected number of deaths according to the existing average mortality data from 2015 to 2019. The shaded area represents the number of cases died of COVID-19 reported worldwide. The red lines represent the estimated excess mortality. The red numbers depict the total excess mortality between January 1, 2020, and December 31, 2021. The data are available at https://www.who.int/data/stories/global-excess-deaths-associated-with-covid-19-january-2020-december-2021/

**Fig. 4:**
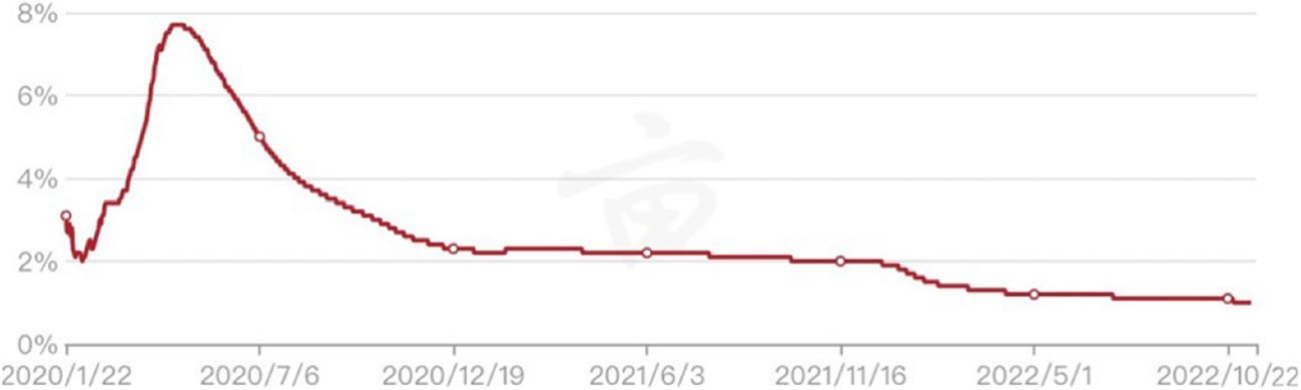
The CRF of COVID-19 in the world as of October 22, 2022. The CFR of COVID-19 increased considerably in the first half of 2020, almost 8%, and declined significantly in the second half of 2020, closing to 2%. Afterwards, the CFR of COVID-19 remained at a relatively stable level of about 2% in 2021, whilst it experienced a further downward trend in 2022 and stabilised at 1% at the end of the year. The data are available at https://coronavirus.1point3acres.com/en

According to WHO, the period from January 1, 2020, to December 31, 2021, witnessed 14.91 million excess mortality related to COVID-19 globally (Fig. 2). The excess mortality in the lower-middle-income World Bank group was 7.87 million, followed by 4.24 million in the upper-middle-income group, 2.16 million in the high-income group and 0.64 million in the low-income group, revealing the impact of income level on COVID-19 response (Fig. 3). As of 4:14 pm CEST on December 31, 2021, the number of confirmed cases has reached 281.81 million including 5.41 million deaths globally [1]. Although the confirmed cases in the middle phase climbed higher and quicker than those in the initial phase, the case fatality rate (CFR) of COVID-19 in 2021 was significantly lower than that in 2020 and remained at a stable level of approximately 2% (Fig. 4).

Most countries in the middle phase began treating COVID-19 as an epidemic instead of a pandemic, and gradually loosening the strict restrictions with the knowledge of COVID-19. One of the representative countries was the USA; due to the overwhelming burden on the health system and economy, they lifted the restrictions and decided to co-exist with COVID-19 on April 2, 2021, allowing citizens to move around freely, whilst the majority of countries were actively promoting vaccination against COVID-19 in response to the epidemic. It was reported that 41.8% of Americans had received the first dose of the COVID-19 vaccine as of April 25, 2021 [15]. Mainland China reported 2.82 billion cumulative vaccinations as of December 31, 2021 [16]. Due to effective prevention and control strategies, the daily new confirmed cases and deaths remained at a relatively stable level with a slight fluctuation in the middle phase [1].

### The current phase (2022–2023)

As of 4:54 pm CEST on 23 December 2022, the number of cumulative confirmed COVID-19 cases has reached 651.92 million including 6.66 million deaths globally [1]. Although most countries had lifted their restrictions and launched the COVID-19 co-existing policy, the CFR did not rebound due to the declined virulence, increased vaccination rate and effective prevention strategies. On the contrary, the CFR further decreased in 2022, remaining stable at 1% (Fig. 4).

The virulence of BA.4/5, the dominant Omicron subvariant in Hong Kong in the phase, was lower than that of previous variants. Under the stepwise loose restrictions in Hong Kong, the number of confirmed, severe and dead cases, and the effective reproduction number (Rt), a measure reflecting the contagiousness in the real world, remained constant [17]. The XBB.1.5 subvariant, an offshoot of SARS-CoV-2 named XBB, made up 61.3% of COVID-19 cases in the USA on January 28, 2023, but the weekly cases and deaths maintained relatively stable [18]. The evidence indicates that Omicron did not pose an increase in the risk of COVID-19 transmission globally.

Numerous studies as to whether COVID-19 increases the risk of sequelae were published in this current phase. A case–control observational study involving 56,003 adults identified that people with an Omicron infection were less likely to experience new or persistent symptoms after infection when compared to delta infection cases (4.5% vs*.* 10.8%) [19]. A Scottish population cohort study with 33,281 infections and 62,957 controls found that there was no association between the asymptomatic and adverse outcomes, and vaccination can decrease the risk of sequela [20]. In addition, a retrospective national cohort study involving 1,913,234 participants revealed that the health risks in patients with mild COVID-19 symptoms will disappear within a year [21]. The evidence indicates a promising picture of living with COVID-19.

Past strict restrictions for COVID-19 have devastated the health system and economy. Some countries gradually opened their doors in an attempt to embrace and live in harmony with COVID-19. In contrast to the passive co-existing with COVID-19 in the USA, on February 21, 2022, Britain proposed active co-existing with COVID-19 via achieving herd immunity. Though the cases infected with COVID-19 in Britain experienced a sharp rise at the beginning of the co-existing policy, confirmed cases and deaths have significantly dropped since July 2022 [1].

**Fig. 5:**
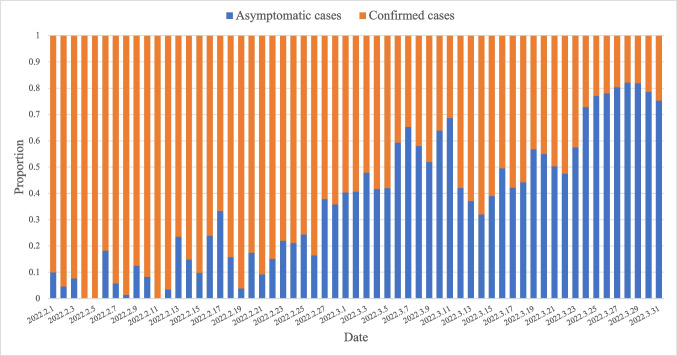
The proportion of daily new residential asymptomatic cases and confirmed cases of COVID-19 in mainland China between February 1 and March 31, 2022. The proportion of asymptomatic cases in mainland China increased considerably from 10% on February 1 to 75% on March 31, 2022, whilst the proportion of confirmed cases in mainland China declined significantly from 90% on February 1 to 25% on March 31, 2022. The data are available at http://www.nhc.gov.cn/xcs/yqtb/list_gzbd.shtml

**Fig. 6:**
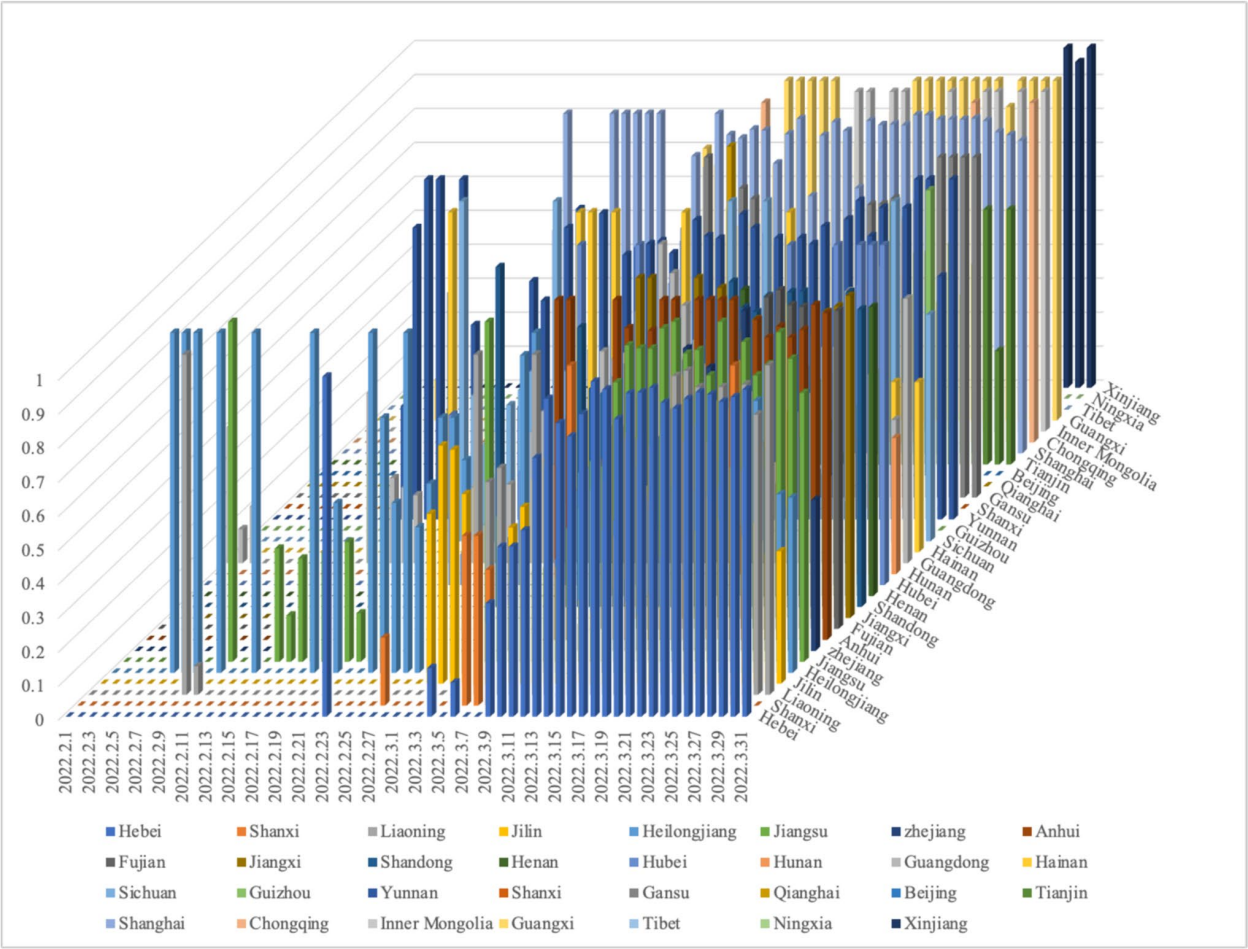
The proportion of new asymptomatic cases of COVID-19 in different provinces of mainland China between February 1 and March 31, 2022. The three-dimensional bar diagram shows the epidemiological differences of COVID-19 in 31 districts in mainland China. Different colour represents different districts. The absence of a bar represents no new asymptomatic cases of COVID-19 on a certain day in a certain district. The data are available at http://www.nhc.gov.cn/xcs/yqtb/list_gzbd.shtml

**Fig. 7:**
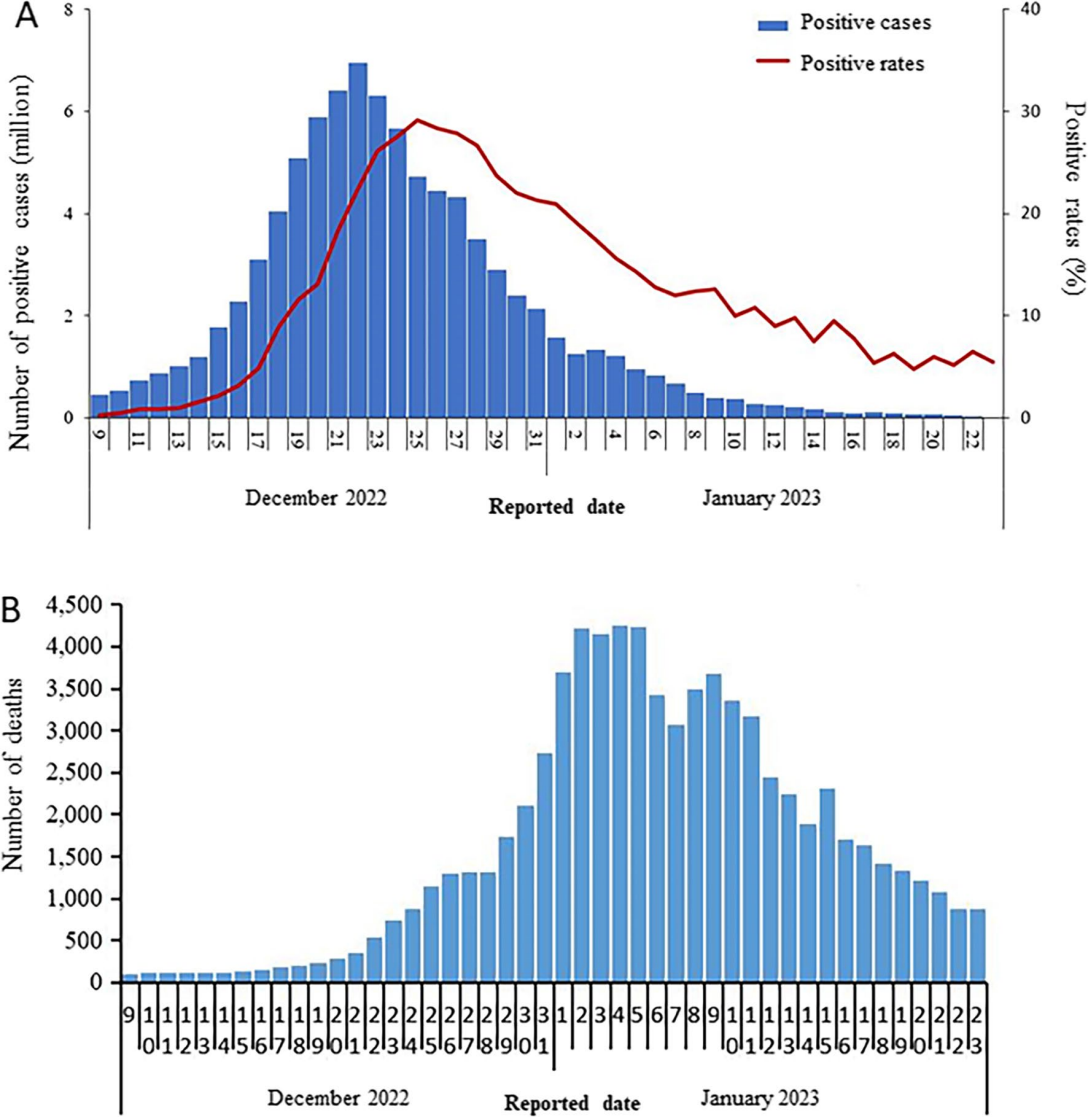
The reported infection from December 8, 2022, to January 23, 2023, after China lifted restrictions. **A** The positive cases and positive rates of COVID-19 determined by nucleic acid testing in China dropped and closed to 0 on January 23, 2023, after experiencing significant growth. **B** The number of deaths in hospitals caused by COVID-19 in China increased dramatically in the first month of lifting restrictions. Afterwards, the number of deaths declined and gradually closed to the situation before lifting restrictions. The data are available at https://www.chinacdc.cn/jkzt/crb/zl/szkb_11803/jszl_13141/202301/t20230125_263519.html

In China, the number of asymptomatic cases of COVID-19 raised greatly due to the declining virulence of COVID-19 and rapid screening in initial and middle phases. For example, the period from February 1 to March 31, 2022, witnessed a significant growth in the proportion of asymptomatic infections in 31 districts in mainland China (Figs. 5 and 6). Since then, the asymptomatic cases in mainland China have reached and maintained over 80% [22]. Subsequently, China completely lifted COVID-19 restrictions on December 7, 2022, according to real-time epidemic conditions, which removed the onerous nucleic acid testing for citizens and cancelled quarantine of confirmed and asymptomatic infections, after having insisted on the strict “dynamic COVID-free strategy” for 3 years. Although the cancelled restrictions led to a new wave of infections, the reported positive rates and in-hospital deaths of COVID-19 have significantly declined and stabilised by the end of January 2023 (Fig. 7). Importantly, this trend did not rebound despite the increase of movement in and around China during the 2023 Chinese New Year. With great hope, WHO declared an end to COVID-19 on May 5, 2023 [3].

### Potential role of PPPM in COVID-19

PPPM is one of the great revolutions in medical history, which is an advanced healthcare approach that aims to provide optimal medical services from predictive approaches, targeted prevention and personalised treatments [4, 23, 24]. Taking primary prevention for healthy individuals, secondary prevention for susceptive populations and tertiary prevention for patients into consideration, PPPM has been successfully applied in diabetes [23], rare diseases [4], reproductive diseases [25] and others. Furthermore, during the COVID-19 pandemic, the concept of PPPM was innovatively applied to the fight against COVID-19 [26–30].

### Predictive models applied during COVID-19

Predicting COVID-19 infection, severity, prognosis and transmission was critical during the outbreak. A diagnostic prediction model has demonstrated effectiveness in excluding COVID-19 infection amongst those with suspected COVID-19 infection, which is important for COVID-19 prevention and control in the hospital setting before nucleic acid testing result is obtained [31]. Meta-analysis revealed that elevated C-reactive protein, interleukin-6 and other inflammatory biomarkers can predict the severity and progression of COVID-19 [32]. A prospective population-based cohort study indicated that a risk model involving age, gender, ethics, comorbidities and other factors showed a good performance in predicting COVID-19-related death and hospitalisation amongst the vaccinated population [33], which is beneficial for the targeted intervention and the deployment of public health policies. In addition, an algorithm to predict the risk of hospitalisation and stratify COVID-19 patients based on data from the Italian population has assisted in the allocation of medical resources [34]. A recursive mathematical model developed to predict virus transmissibility based on public data worldwide has evaluated the effectiveness of the quarantine strategies and provided guidance for health policy making [35].

Furthermore, continually monitoring the new variants and their virulence and adapting strategies accordingly are helpful to prevent COVID-19 spread under predictive and preventive medicine. Currently, the main variant of SARS-CoV-2 that circulated worldwide is Omicron, which consists of over 98% viral sequences deposited in the Global Initiative on Sharing All Influenza Data (GISAID), a publicly available database providing genomic and associated metadata of influenza viruses. A model based on the interaction of the SARS-CoV-2 spike and angiotensin-converting enzyme 2 (ACE2) can predict the binding affinity of SARS-CoV-2 variants and ACE2, as well as predict the transmissibility and virulence of the new variants, proving a powerful tool for new vaccine development and application of PPPM in pandemic control [36].

### Three levels for targeted prevention of COVID-19

We proposed three levels of COVID-19 prevention and management by reference to the three levels of application of PPPM for chronic diseases, such as diabetes care [4].

The first level is to protect healthy people from infection during the pandemic. For example, education on practicing hand hygiene, wearing a mask, keeping physical distance, avoiding large gatherings, especially in enclosed spaces, and receiving vaccine as soon as possible is necessary to reduce the risk of becoming infected with the SARS-CoV-2. In addition, mass community screening by nucleic acid testing is effective to avoid the spread of COVID-19 through early recognition of positive patients [8].

The second level is to protect the susceptible or vulnerable individuals with poor resistance and immunity from infection, such as seniors, children and patients with chronic non-infectious disease. For example, mass vaccination covering people over 60 years old or children under 14 years old contributes to protecting vulnerable people from catching severe COVID-19. According to the official data from the Chinese Center for Disease Control and Prevention [37], a total of 3491 billion doses of vaccination with a coverage of 92.9% of the first dose and 90.6% of the third dose have been completed as of February 6, 2023. Particularly, those who received the first dose and the third dose accounted for 96.6% and 92.2% of the people over 60 years old, respectively. Furthermore, providing comprehensive professional medical care is essential for those who suffered from chronic disorders, e.g. chronic liver disease, during the COVID-19 pandemic [38].

The third level is to isolate and treat people infected with COVID-19. Those suffering from COVID-19 infection are suggestive to seek medical attention or be quarantined at home and avoid contact with others as much as possible. Paying more attention to secondary complications of COVID-19 patients and providing them with tailored treatment are another matters worthy of note. Pneumomediastinum, kidney infarction, splenic infarction, ischemic stroke, thrombosis and hematomas are common secondary complications of COVID-19 [39]. In addition, three cases complicated with Takotsubo syndrome due to acute stress after being infected with COVID-19 were reported [40].

### Personalised strategies in response to COVID-19

Since COVID-19 was first reported in Wuhan, China, in 2019, countries have applied distinct policies and measures in response to the outbreak based on the severity of the pandemic and national condition. For example, the governments of the USA, Australia, Britain and other Western countries developed herd immunity to achieve co-existing with COVID-19, allowing for social and economic activities to continue, whilst the Chinese government applied the personalised COVID-free policy to minimise infections and deaths when considering the large national population base and density. Later on, China completely lifted the restrictions and cancelled the mass nucleic acid screening on December 7, 2022, owing to the dropped CFR, variants with low virulence, an increased proportion of asymptomatic infections and decreased risk of sequelae.

The personalised strategies in response to COVID-19 contributed to a lower CFR [41]. As the cases with mild to moderate COVID-19 were quarantined at home, more medical services were concentrated on the severe and critical patients. This measure was conducted by the majority of countries, resulting in fewer resource costs outlaid and being able to aid in the most critical patients’ survival. It was identified that the global CFR, due to COVID-19, considerably declined to 1.0% on October 22, 2022 (Fig. 4). Therefore, the dynamic personalised policies, as well as real-time prevention and control approaches based on the epidemic situation, helped contain the deterioration of the CFR during the pandemic.

## Data interpretation

### The COVID-19 pandemic emergency was declared over

Currently, epidemiological characteristics show that the trends of the confirmed cases, deaths, CFR and virulence of COVID-19 have decreased, and the coverage of vaccination has increased in the current phase, compared with the initial and middle phases of COVID-19 [1]. Less risk of sequelae was reported in several landmark large-scale studies [19–21]. In addition, the application of PPPM concept provided some guidance on the prediction, prevention and formulation of personalised strategies in response to COVID-19 [8]. On January 30, 2023, WHO declared that COVID-19 was probably at a transition point from a PHEIC to an end [42]. On April 4, 2023, Joe Biden, the president of the USA, signed a bill to end the COVID-19 national emergency [43]. On May 5, 2023, WHO officially declared an end to COVID-19 as a global health emergency [3].

### Lessons drawn from fighting against COVID-19

CFR is defined as a proportion of cases who died of a particular disease amongst the infectious cases during a period [44]. Serving as an indicator of the success of preventive measures, CFR reflects whether the strategies are effective in reducing the incidence and severity of COVID-19. A low CFR may decrease unnecessary social concern and fear, avoid overburdened health systems, balance the incorrect allocation of medical resources, and prevent radical prevention and control strategies. However, this calculation sometimes is misleading in certain conditions because it considers the deaths after infection as the cases died of infection. For example, patients who died of COVID-19 from advanced cancer or patients over 90 years old who died of COVID-19 are counted in CFR. Actually, these people experience a high risk of death irrespective of COVID-19, which causes an overestimation of the COVID-19 CFR [45]. Therefore, the actual CFR in every country is lower than the currently reported in the real-world scenario, which should be fully recognised. In addition, no one can regulate COVID-19 from a political perspective; instead, we should manage the challenge of PHEIC from a scientific perspective based on Koch’s postulates of infectious disease, a set of criteria proposed by Robert Hermann Koch in 1890 to clarify the causality between microbe and disease [46]. The source of infection, route of transmission and susceptible population are three aspects that should be emphasised.

### The paradigm shift from reactive medicine to PPPM

The European Association for Predictive, Preventive and Personalised Medicine (EPMA), created in 2009, has made considerable efforts in the paradigm change from reactive medicine to PPPM [24], being partly reflected in the fight against COVID-19. Firstly, the predictive approach in PPPM involves the use of advanced technologies and intelligent algorithms to identify potential health risks and predict the onset of diseases, which makes medical professionals take a proactive approach to prevent diseases before they occur. For example, the autoverification and guidance system, a computer-based intelligent procedure, was applied to rapidly diagnose COVID-19 with a high accuracy [8]. Secondly, the targeted prevention enables medical professionals to intervene individuals who are at the highest risk of developing specific diseases. Finally, the personalisation of medical services involves tailored medical care to individuals based on their unique characteristics and needs, on top of the standardised treatment derived from guideline recommendations. For example, a dental care scheme based on specific age group was established during the COVID-19 pandemic, aiming to provide targeted prevention and tailored treatment for dental diseases [47]. However, devastating outcomes of COVID-19 demonstrated that PPPM principles are currently not implemented widely and timely but have to be introduced into daily practice as soon as possible.

### PPPM attitude was not adopted early in the COVID-19 pandemic

Although the whole concept of PPPM was already known and well promoted when the COVID-19 was declared in January 2020 a worldwide public health emergency, the factual role of PPPM was limited in fighting against COVID-19. This may be one of the reasons why COVID-19 has persisted for more than 3 years. Therefore, a thorough understanding and an early application of PPPM to maximise its pivotal role in fighting against COVID-19 and future infectious disease are highly beneficial to end the pandemic in advance. Furthermore, PPPM warns us that medical resources should be allocated rationally between the prevention of infectious disease such as COVID-19 and the management of non-infectious diseases, which would help reduce the excess mortality resulting from the COVID-19 pandemic.

## Conclusion and expert recommendations

In this review, we first described the international situation and the prevalence of COVID-19 globally in three phases: initial (2019–2020), middle (2021) and current (2022–2023), based on the public database and bibliometric analysis. Subsequently, we analysed the potential role of PPPM in fighting against COVID-19, including (1) various predictive models in diagnosing COVID-19 infection, predicting the severity and prognosis of infected cases, and the transmission of SARS-CoV-2; (2) three levels focusing on three stratified populations in preventing COVID-19, i.e. education of uninfected healthy population, protection of susceptible individuals, and isolation and treatment of infected patients; and (3) personalised policies and measures of COVID-19 prevention and control. Lastly, we concluded the lessons learned from the process of dealing with COVID-19 and proposed our viewpoints and suggestions for facing future infectious diseases.

The application of PPPM improves our understanding of infectious disease and awakes us to remain vigilant and adopt the PPPM measures during the ongoing evolution of a pandemic. When formulating, reformulating and implementing strategies and mechanisms for the prevention and control of infectious disease, considering the changing situation and giving introspective thought are the key. Under the current epidemic situation, early and widely applying predictive models (e.g. predict infection, severity, prognosis and transmission), targeted prevention and dynamic personalised prevention and control policies as well as individual treatments would further mitigate the loss caused by infectious disease considerably. In summary, we firmly believe that COVID-19 and similar pandemics or PHEIC in the future will transform from a pandemic into an endemic phase and conclude sooner whilst PPPM is implemented early, widely and adequately in prevention and control, along with the application of efficient vaccination and invention of effective viral therapy. COVID-19 is still considered a global threat though the end of COVID-19 has been declared. We have to learn from this experience to be able to face the similar situation more effectively and more wisely in the future.

## Data Availability

The dataset generated and analysed during the study are available from the corresponding author on reasonable request.

## Abbreviations

ACE2: Angiotensin-converting enzyme 2
CFR: Case fatality rate
COVID-19: Coronavirus disease 2019
GDP: Gross domestic product
GISAID: Global Initiative on Sharing All Influenza Data
PHEIC: Public Health Emergency of International Concern
PPPM: Predictive preventive personalised medicine
Rt: Reproduction number
SARS-CoV-2: Severe acute respiratory syndrome coronavirus 2
WHO: World Health Organization
